# BAG9 Confers Thermotolerance by Regulating Cellular Redox Homeostasis and the Stability of Heat Shock Proteins in *Solanum lycopersicum*

**DOI:** 10.3390/antiox11081467

**Published:** 2022-07-27

**Authors:** Huamin Huang, Chenxu Liu, Chen Yang, Mukesh Kumar Kanwar, Shujun Shao, Zhenyu Qi, Jie Zhou

**Affiliations:** 1 Department of Horticulture, Zhejiang Provincial Key Laboratory of Horticultural Plant Integrative Biology, Zhejiang University, Yuhangtang Road 866, Hangzhou 310058, China; 22016059@zju.edu.cn (H.H.); chenxuliu@zju.edu.cn (C.L.); 20210107@hznu.edu.cn (C.Y.); kanwar@zju.edu.cn (M.K.K.); ssjun@zju.edu.cn (S.S.); 2 Hainan Institute, Zhejiang University, Sanya 572025, China; qizhenyu@zju.edu.cn; 3 Agricultural Experiment Station, Zhejiang University, Hangzhou 310058, China; 4 Key Laboratory of Horticultural Plants Growth, Development and Quality Improvement, Ministry of Agriculture and Rural Affairs of China, Yuhangtang Road 866, Hangzhou 310058, China; 5 Shandong (Linyi) Institute of Modern Agriculture, Zhejiang University, Linyi 276000, China

**Keywords:** antioxidants, BAG9, Hsps, *Solanum lycopersicum*, thermotolerance

## Abstract

The Bcl-2-associated athanogene (BAG) family, a group of co-chaperones that share conservative domains in flora and fauna, is involved in plant growth, development, and stress tolerance. However, the function of tomato *BAG* genes on thermotolerance remains largely unknown. Herein, we found that the expression of *BAG9* was induced during heat stress in tomato plants. Knockout of the *BAG9* gene by CRISPR/Cas9 reduced, while its overexpression increased thermotolerance in tomato plants as reflected by the phenotype, photosynthesis rate, and membrane peroxidation. Heat-induced reactive oxygen species and oxidative/oxidized proteins were further increased in *bag9* mutants and were normalized in *BAG9* overexpressing plants. Furthermore, the activities of antioxidant enzymes, ascorbic acid (AsA)/dehydroascorbic acid (DHA), and reduced glutathione (GSH)/oxidized glutathione (GSSG) were reduced in *bag9* mutants and were increased in *BAG9* overexpressing plants under heat stress. Additionally, BAG9 interacted with Hsp20 proteins in vitro and in vivo. Accumulation of Hsp proteins induced by heat showed a reduction in *bag9* mutants; meanwhile, it was increased in *BAG9* overexpressing plants. Thus, BAG9 played a crucial role in response to heat stress by regulating cellular redox homeostasis and the stability of heat shock proteins.

## 1. Introduction

Global warming exacerbates the occurrence of extreme weather, among which high temperature is a major environmental threat to crop yields [1]. Under heat stress, the ultrastructure and function of chloroplasts and mitochondria suffer damage, resulting in a burst of reactive oxygen species (ROS), such as singlet oxygen, superoxide anion, hydrogen peroxide, and hydroxyl [2]. The accumulation of ROS leads to the damage of nucleotides, membrane lipid peroxidation, and protein denaturation [3,4]. Furthermore, protein denaturation induced by high temperature results in oxidation, misfolding, and aggregation of proteins. The gathering of these proteins leads to cell death in the absence of chaperones, proteasomes, and autophagy systems [5].

Molecular chaperones help in maintaining protein homeostasis under heat by restoring the native conformation of proteins and preventing the aggregation of non-native proteins for later folding or assembling [6]. Five groups of molecular chaperones heat shock proteins (Hsps) have been identified, including small heat shock proteins (sHsps)/Hsp20, Hsp60, Hsp70, Hsp90, and Hsp100 [7,8]. They not only protect proteins, but also increase the stability of lipid membranes, membrane proteins such as the photosystems, and soluble proteins [9]. Small Hsps are distinguished from other Hsps since they work in an ATP-independent manner to form a complex with non-native proteins preventing the harmful aggregation of proteins under stress [10]. Overexpressing *OsHsp18.2* in *Arabidopsis* highly enhanced the activity of seeds and the percentage of germination under heat stress [11]. Hsp60 especially improved the thermotolerance of plastid proteins such as Rubisco and retarded cell death [12,13]. Hsp90 interacted with the FK506 binding proteins (FKBPs) regulating thermotolerance [14]. In Hsp100 class, Hsp101 exhibited significant heat resistance and functioned well in recovery from heat shock [15,16]. Co-operation between Hsp100 and heat stress-associated 32-KD protein (HSA32) promoted the effects of heat acclimation in rice seedlings [17].

Among Hsps, Hsp70 regulating mechanism has been widely researched [18]. The work of hsp70 is assisted by a large chaperone system [19,20,21]. Under cell stress, ATP hydrolysis is indispensable for the binding of Hsp70 to polypeptide chains in non-native protein structures [22]. J-proteins are significant components in the Hsp70 chaperone system, which involve in heat stress response by regulating ATP activity, thus enhancing the binding affinity of Hsp70 with unfolded peptides or other substrates [23]. Nucleotide exchange factors (NEFs) are also necessary co-chaperones in the Hsp70 system [24]. Bcl-2-associated athanogene (BAG) has been identified as a NEF chaperone family, which contains a BAG domain interacting with Hsp70 on its ATPase domain, influencing nucleotide exchange by assisting ATP to bind with Hsp70 and releasing ADP, enhancing protein quality control. The BAG family may establish an association between the Hsp chaperone system and its substrates [25].

As chaperones, the BAG family in plants plays various roles in response to multiple stresses such as heat, freezing, salinity, drought, and ultraviolet (UV) [26,27]. For temperature resistance, *Atbag2* or *Atbag6* mutants survived worse under heat [28]. Upon sensing heat, the processed AtBAG7 entered the nucleus from the endoplasmic reticulum (ER) to interact with WRKY29, initiating unfolded protein response (UPR) pathway to enhance thermotolerance [29,30]. For pathogen resistance, BAG6 activated autophagy by being cleaved by aspartyl protease (APCB1) upon recognizing an intrusive pathogen in *Arabidopsis thaliana* [31]. Similarly in rice, enhanced blight and blast resistance 1 (EBR1) targeted OsBAG4, ubiquitinating and degrading it for immunity regulation and extensive defense against disease [32]. For inhibiting senescence, the signal complex calmodulin-like motif (CaM)/AtBAG5/heat shock cognate 70 (Hsc70) upregulated a high level of Ca^2+^ in mitochondria to inhibit senescence [33]. Likewise in tomato, BAG2 and BAG5b improved the resistance to dark-induced leaf senescence [34]. Various abiotic stresses induced AtBAG4 and regulated ion channels and stomatal motion by interacting with and adjusting KAT1 [35,36].

Tomato is one of the main economic crops in protected cultivation. Heat stress deranges metabolic imbalance in tomato, highly decreasing the quality and production [37]. However, the mechanism of BAGs affecting the thermotolerance of tomato is unclear. To further explore the role of the BAG chaperone family under heat stress and its relationship with Hsps, we generated *BAG9* overexpressing lines and *bag9* mutants and treated them with high temperature. We observed the phenotypes and measured a range of resistance indicators. Results showed that *bag9* was more sensitive to heat stress compared to the wild type (WT), while *BAG9* overexpressing plants showed the opposite tendency. It indicated a positive regulatory effect of BAG9 in temperature tolerance.

## 2. Materials and Methods

### 2.1. Phylogenetic Analysis and Structural Domain Prediction of BAG Family

The amino-acid sequences of BAG family proteins in *Solanum lycopersicum*, *Arabidopsis thaliana*, *Oryza sativa*, and *Nicotiana tabacum* were obtained from the Ensembl Plants database (http://plants.ensembl.org (accessed on 4 May 2020)). The set of protein sequences was imported into the Molecular Evolutionary Genetics Analysis tool (MEGA 11) and multiplexed using the ClustalW method and exported in MEGA format. The phylogenetic tree was constructed using the maximum likelihood tree (ML) method and the bootstrap analysis was applied with 1000 replicates/iterations. Finally, the constructed phylogenetic tree was polished with Evolview (http://evolgenius.info (accessed on 5 May 2020)). Structural domains of the BAG family in tomato were analyzed using the native InterProScan program (http://www.ebi.ac.uk/interpro/ (accessed on 5 May 2020)). The structural domain sequences were obtained from the Pfam database and the structural schematics were manufactured using Domain Graph (DOG) software (http://dog.biocuckoo.org/ (accessed on 5 May 2020)).

### 2.2. Plant Material, Growth Condition, and Heat Treatment

Ailsa Craig (AC) of tomato from Tomato Genetics Resource Center (TGRC) was used as a wild type (WT). Peat and vermiculite were mixed in a suitable ratio (7:3, v:v) for seedling growth. Hoagland nutrient solutions were used twice a week to supplement the tomato with nutrients. The growing conditions of plants were ensured according to the following criteria: photoperiod was performed by 14 h/10 h (day/night), the ambient air temperature was kept by 25 °C/20 °C (day/night), and photosynthetic photon flux density was arranged to 400 μmol m^−2^ s^−1^. Plants at the five-week seedling stage were used for the following experiments. Two groups of AC, OE-*BAG9*, and *bag9* plants were separated. The control group and the heat stress group were treated for 10 h at 25 °C and 45 °C, respectively, in growth chambers (Qiushi, Hangzhou, China). Except for the temperature in the growth chambers, other environmental parameters remained the same as previously described. Leaf samples were collected at different times from heated or unheated tomato plants, then frozen rapidly in liquid nitrogen and stored at −80 °C before analysis for gene expression, malondialdehyde (MDA), antioxidant, enzyme activity, and immunoblotting. While after being treated for 7 h, leaf samples were collected from the control group and the heat stress group and then immediately analyzed for a maximum quantum yield of PSII (*Fv*/*Fm*) and 3,3′-diaminobenzidine (DAB) and nitroblue tetrazolium (NBT) staining.

### 2.3. Construction of Plant Expression Vector and Tomato Transformation

To generate the *BAG9*-overexpressing lines, *BAG9* full-length coding sequence (CDS) was amplified with the forward primer (5′-gggcgcgccgatatcgtcgacATGGAGAATCTCTTCAATTGGTCC-3′) and reverse primer (5′-aacatcgtatgggtaggtaccGCTGCCGGAAACAATGGAG-3′) using tomato complementary DNA (cDNA) as the template. To insert the PCR product into the pFGC1008-HA vector behind the cauliflower mosaic virus (CaMV) 35S promoter, the product was digested with *Asc*I and *Kpn*I. As described previously, CRISPR/Cas9 vectors were constructed and used to generate *bag9* mutants [38]. Using the CRISPR-P web tool (http://crispr.hzau.edu.cn/ (accessed on 11 September 2020)), the target sequences (5′-GCTCGCCGTCGCTATTCCTC-3′) were achieved and subsequently introduced into the *Bbs*I site of the AtU6-sgRNA-AtUBQ-Cas9 vectors following annealing into the double strands. The fragments of the AtU6-sgRNA-AtUBQ-Cas9 were fused to the *Kpn*I and *Hind*III sites of the pCAMBIA1301 binary vectors. The final vectors were introduced into tomato AC via *A. tumefaciens*-mediated transformation. A homozygous T2 *BAG*9 overexpressing line was used for experiments and identified by Western blot using an anti-HA (26183, Thermo Fisher Scientific, Waltham, MA, USA) monoclonal antibody (Figure S1A). *bag9* mutant contained mutations near the protospacer adjacent motif (PAM), which induced mismatched amino-acid sequence and terminated translation (Figure S1B). 

### 2.4. Total RNA Extraction and Gene-Expression Analysis

RNA extraction kits were used for obtaining total RNA (DP419, Tiangen, Beijing, China). The HiScript Q RT SuperMix for the quantitative real-time PCR (+gDNA wiper) Kit (R223, Vazyme, Nanjing, China) was used to produce first-strand cDNA from 500 ng of total RNA. ChamQ Universal SYBR qPCR Master Mix (Q711, Vazyme, Nanjing, China) and Light Cycler^®^ 480 II Real-Time PCR detection system (Roche, Basel, Switzerland) were used in the RT-qPCR. In this program, predenaturation at 95 °C for 3 min, followed by 40 cycles of denaturation at 95 °C for 30 s, annealing at 58 °C for 15 s and 72 °C for 30 s, and a final extension at 72 °C for 30 s. Table S2 listed primers used for RT-qPCR, as well as tomato *Actin* as an internal control. To calculate relative gene expression, the 2^−∆∆CT^ method was used, and a heat-map analysis was conducted using MEV version 4.9 (http://www.mev.tm4.org/ (accessed on 10 June 2020)). At the bottom, the intensity of the color bar showed the intensity of expression.

### 2.5. Gas Exchange and Chlorophyll Fluorescence Measurements

The infrared gas analyzer-based portable photosynthesis system (LI-6400T, Li-Cor Inc., Lincoln, NE, USA) was applied for measuring the net photosynthetic rate (*P*n) in plants under heat or controlled environment. The measurements were carried out at 1000 µmol m^−2^ s^−1^ photosynthetic photon flux density (PPFD), 400 µmol mol^−1^ atmospheric carbon dioxide (CO_2_) concentrations, and 25 °C leaf temperature, respectively. Fluorescence measurements for chlorophyll were conducted using a MAXI Version of the Imaging-PAM M-Series fluorescence system (Heinz-Walz, Effeltrich, Germany). For 30 min prior to measurement, plants were kept in the dark. According to previous descriptions, the maximum quantum yield of PSII (*Fv/Fm*) was measured and calculated [39]. 

### 2.6. Analysis of H_2_O_2_, O_2_^•−^ and Malondialdehyde (MDA)

In order to observe the accumulation of hydrogen peroxide (H_2_O_2_) and superoxide anion (O_2_^•−^) on leaves, the DAB and NBT staining were performed as previously described with minor modifications [40]. 

For O_2_^•−^ staining, leaf samples were stained with 0.5 mg mL^−1^ NBT in 25 mM N-2-hydroxyethylpiperazine-N-ethane-sulphonic acid (HEPES) (pH 7.8) and incubated in the dark under 25 °C for 6 h. For H_2_O_2_ staining, leaf samples were stained with 1 mg mL^−1^ DAB in 50 mM Tris-HCl (pH 3.8) and incubated at 25 °C for 12 h in the dark. In both cases, leaf samples were washed in 95% (v:v) ethanol for 10 min at 95 °C, kept in lactic acid/phenol/water (1:1:1; v:v:v), and photographed.

The H_2_O_2_ concentration in the leaves was quantified based on the method described previously with minor modifications [41]. In brief, a 0.3 g leaf sample was taken for analysis. After being ground with 3 mL 0.2 M HClO_4_ in liquid nitrogen, the material was centrifuged at 6000 g for 10 min at 4 °C. A total of 4 M KOH was used to neutralize the pH to about 6–7. 0.05 g activated carbon was added and the solution was centrifuged at 12,000× *g* for 5 min at 4 °C. The 0.22 μm filter membrane was used to filter the supernatant into a new centrifuge tube to obtain extracting solution. A total of 100 mM potassium acetate buffer (pH 4.4, containing 1 mM ABTS) was used as the reaction buffer. For the nonenzymatic tube reaction system, 1 mL H_2_O_2_ sample and 1 mL reaction buffer were mixed and the absorption peak at 412 nm was determined. For the enzyme tube reaction system, 1 mL H_2_O_2_ sample, 996 μL reaction buffer, and 4 μL horseradish peroxidase (POD) were mixed. Finally, the absorption peak at 412 nm was determined to measure the content of H_2_O_2_. The content of MDA in the leaves was measured according to a previous protocol [39]. Extracted leaves were heated at 95 °C for 25 min with trichloroacetic acid containing 0.65% 2-thiobarbituric acid (TBA). By subtracting the absorbance at 532 nm of a TBA-free solution containing the plant extract, non-MDA compounds were corrected.

### 2.7. Antioxidant and Enzyme Activity Assays

For nonenzymatic antioxidant assays, approximately 100 mg of leaf sample was powdered in liquid nitrogen and extracted into 1 mL 0.2 M HCl. The solution was centrifugated by 12,000 g for 10 min under 4 °C and then 0.2 M NaOH was used to neutralize the mixed solution to pH 4–5 containing 500 μL supernatant of the last step and 100 μL 0.2 M phosphate buffer (pH 5.6). Finally, spectrophotometric assays were used to measure the extracting solution for ascorbic acid (AsA)/dehydroascorbic acid (DHA), and reduced glutathione (GSH)/oxidized glutathione (GSSG) according to previous methods [42].

To measure antioxidant enzyme activity, 300 mg leaf sample was milled with 3 mL of ice-cold enzyme buffer containing 25 mM HEPES, 0.2 mM ethylene diamine tetraacetic acid (EDTA), 2 mM AsA, and 2% polyvinylpolypyrrolidone (w:v) (pH 7.8). The extracting solution was centrifugated at 12,000× *g* for 10 min under 4 °C and then the supernatants were kept for measurement. Subsequently, SHIMADZU UV-2410PC spectrophotometer (Shimadzu, Kyoto, Japan) was employed to detect enzyme activity. The activities of antioxidant enzymes catalase (CAT), ascorbate peroxidase (APX), glutathione reductase (GR), and dehydroascorbate reductase (DHAR) were analyzed according to the previous protocol with minor modifications [43]. For analyzing CAT activity, 100 μL of enzyme solution, 1700 μL of 25 mM phosphate buffer Solution (PBS) (PH 7.0, containing 0.1 mM EDTA), and 200 μL of 100 mM hydrogen peroxide were mixed. The kinetic changes of OD240 were determined according to the kinetic program, and the enzymatic reaction rate was calculated by taking the kinetic changes of 10 s. For analyzing APX activity, 100 μL of enzyme solution, 1700 μL of 25 mM PBS (pH 7.0, containing 0.1 mM EDTA), 100 μL of 20 mM H_2_O_2_, and 100 μL of 5 mM AsA were mixed together at 25 °C. The kinetic changes of OD290 were determined according to the kinetic program, and the enzymatic reaction rate was calculated by taking the kinetic changes of 10 s. The reaction rate without H_2_O_2_ was used as blank control. For analyzing GR activity, 100 μL of enzyme solution, 1700 μL of 25 mM PBS buffer (PH7.8, containing 0.2 mM EDTA), 100 μL of 10 mM GSSG, and 100 μL of 2.4 mM NADPH were mixed together at 25 °C. The kinetic changes of OD340 were measured, and the enzymatic reaction rate was calculated by taking the kinetic changes of 10 s. For analyzing DHAR activity, 100 μL of enzyme solution, 1700 μL of 25 mM PBS (pH 7.0, containing 0.1 mM EDTA), 100 μL of 70 mM GSH, and 100 μL of 8 mM DHA were mixed together. The kinetic changes of OD265 were measured, and the kinetic change of 10 s was taken to calculate the enzymatic reaction rate. The enzyme activities of superoxide dismutase (SOD) and peroxidase (POD) were detected according to the previous protocol with minor modifications [44]. For analyzing SOD activity, 50 μL of enzyme solution and 3 mL reaction solution (containing 50 mM PBS (pH 7.8), 15 mM methionine, 65 mΜ NBT, 2 μM riboflavin, 0.1 mM EDTA) were mixed. After 15 min illumination at 25 °C, 4000 lx, the absorbance was measured at 560 nm. For analyzing POD activity, 100 μL of enzyme solution, 1700 μL of 25 mM PBS (pH 7.0, containing 0.1 mM EDTA), 100 μL of 10 mM H_2_O_2_, and 100 μL of 1% guaiacol were mixed together at 25 °C. The kinetic changes of OD470 were determined according to the kinetic program, and the kinetic changes of 10 s were taken to calculate the enzymatic reaction rate.

### 2.8. Immunoblotting Assay

Following the manufacturer’s instructions, the oxidized protein fractions extracted from the soluble protein were tested with an OxyBlot Protein Oxidation Detection Kit (Chemicon International, Temecula, CA, USA). 

For immunoblotting assay, the protein extraction and Western blotting assay were modified by protocol described previously [45]. A 0.1 g leaf sample was grinded in liquid nitrogen and added with the extraction buffer (100 mM Tris-HCl, pH 8.0, 10 mM NaCl, 1 mM EDTA, 1% Triton X-100, 1 mM phenylmethylsulphonyl fluoride, and 0.2% β-mercaptoethanol). The Bio-Rad protein assay kit was used to measure the protein concentration and the total protein concentration of all samples were adjusted to 6 μg/μL. After denaturation by 95 °C for 10 min, the protein samples were detected by sodium dodecyl sulfate-polyacrylamide gel electrophoresis (SDS-PAGE) and were subsequently transferred to nitrocellulose membrane (GE Healthcare Biosciences, Piscataway, NJ, USA). Antibodies of cytosolic Hsp90 (AS08 346, Agrisera, Vännäs, Sweden), Hsp70 (PHY0034S, Phytoab, San Jose, CA, USA), Hsp101 (AS07 253, Agrisera, Vännäs, Sweden) and Hsp17.6 (PHY0149S, Phytoab, San Jose, CA, USA) were used to detect proteins. Afterwards, the goat anti-rabbit horseradish peroxidase-linked antibody (7074, Cell Signaling Technology, Boston, MA, USA) was used as the secondary antibody for these analyses.

### 2.9. Yeast Two-Hybrid (Y2H) Screen and Assays, and Bimolecular Fluorescence Complementation (BiFC) Assay

In order to find out BAG9-interacting proteins in tomato, the coding sequences of *BAG9* were cloned into the pGBKT7 vector using gene-specific promoters (Table S3) and subsequently transferred into the AH109 yeast strain. The cDNA library building and Y2H screening were implemented as the manufacturer’s protocol described (Takara, Shiga, Japan). SD-Trp-Leu-Ade-His plates were used for Y2H screening. Hsp20s in tomato were identified as BAG9-interacting proteins from Y2H screens. The coding sequences of Hsp20s were amplified by PCR using specific primers (Table S4) and cloned into a pGADT7 vector. Cotransformed bait-and-prey constructs were plated onto a selection medium lacking Trp, Leu, Ade, and His to analyze interactions. Before this study, pFGC-N-YFP and pFGC-C-YFP had been described for the BiFC vectors [46]. Gene-specific primers were used to amplify the full-length sequences of BAG9 and Hsp20s in PCRs and clone them into pFGC-N-YFP or pFGC-C-YFP vectors (Figure S5). To infiltrate *N. benthamiana*, plasmids were infectively introduced into *A. tumefaciens* GV3101 strains, according to previously described procedures [46]. During 48 h after infiltration, fluorescent signals from infected tissues were analyzed by a Zeiss LSM 780 confocal microscope (Zeiss LSM 780, Oberkochen, Germany) using appropriate filter sets (excitation wavelengths 488 nm and emission between 500 nm and 530 nm).

### 2.10. Statistical Analysis

Each determination was repeated at least three times independently. Based on the results of independent biological replicates, the data were presented as means ± standard deviations. Analyzing the bioassays was accomplished using SPSS 25 statistics 25 (SPSS Inc., Chicago, IL, USA). In the analysis of treatment differences, Tukey’s test was used at 0.05 for significance.

## 3. Results

### 3.1. Identification of BAG Homologs in Plants

**Figure 1:**
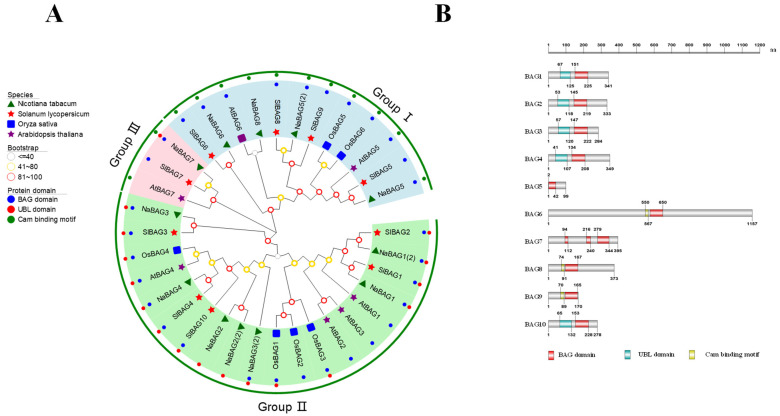
Phylogenetic tree construction of BAGs from different plants and protein structures of BAGs. (**A**) Phylogenetic tree of BAG proteins. The different colored circles on the outside of the protein names represented the types of structural domains possessed by amino-acid sequences (blue for BAG domains, red for UBL domains, and green for calmodulin-binding (CaM) motifs). The symbols on the inside of the protein names represented different species (purple stars for *Arabidopsis thaliana*, green triangles for *Nicotiana tabacum*, blue squares for *Oryza sativa*, and red stars for *Solanum lycopersicum*). (**B**) Schematic diagram of the domains of BAG proteins in tomato. The protein lengths were shown in grey.

Previous studies have demonstrated that the BAG protein family was evolutionarily conserved and highly similar in structure and function in eukaryotes [26]. Phylogenetic analysis of the *BAG* gene family across species was significant for understanding the differences in function or predicting similarities between tomato and other species. We identified 10 *BAG* genes in the tomato genome using the SGN database (https://solgenomics.net/ (accessed on 19 April 2020)) and named *BAG1-10* based on homology and evolutionary analysis with the *Arabidopsis* protein sequences (Figure 1A, Table S1). In light of the function of BAGs, we performed a phylogenetic analysis of BAG proteins from three dicot plants, *Arabidopsis*, tomato, and tobacco (*Nicotiana tabacum*), and a monocot plant, rice. Based on the resultant phylogenetic tree, the BAG proteins of the four species were divided into three subfamilies (Figure 1A). BAG5, BAG6, BAG8, and BAG9 belonged to the first group, BAG7 belonged to the second group, and BAG1, BAG2, BAG3, BAG4, and BAG10 belonged to the third group. 

Then, we further analyzed the structural domains of the BAG proteins (Figure 1B). Results showed that all BAG proteins contained a conservative BAG domain. Furthermore, BAG1-4 and BAG10 contained extra ubiquitin-like (UBL) structural domains at the N-terminus, while BAG6, BAG8, and BAG9 each comprised an extra CaM-binding motif. In addition, BAG7 protein was distinguished since it had no other kinds of motifs but triple BAG domains. In terms of the length of BAG proteins, BAG5 was the shortest, while BAG6 had the longest sequence length.

### 3.2. Involvement of BAG9 in Tomato Thermotolerance

Transcript analysis of 10 *SlBAGs* under heat stress was conducted to determine whether heat stress induced BAG gene expression.

**Figure 2:**
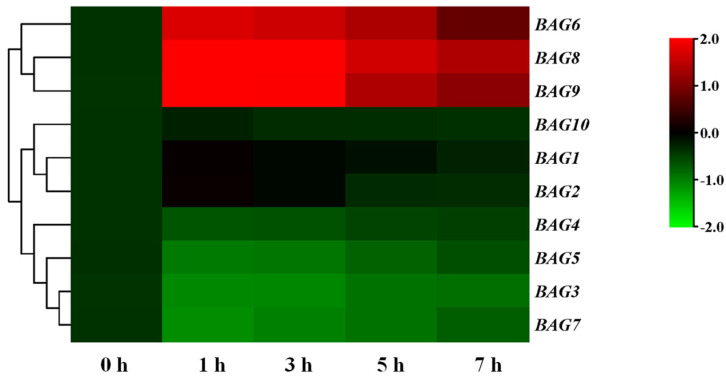
Transcripts of *BAG* genes in response to heat stress. Cluster analysis of expression patterns of *BAGs* under heat stress at 0 h, 1 h, 3 h, 5 h, and 7 h. The heat map was manufactured using log_2_ logarithmic-transformed expression values. The color transition from red to green on behalf of high to low expression levels. According to the expression, the *BAGs* were clustered in the figure. The data represented the means ± SD of three biological replicates.

Figure 2 displayed that exposure to heat within 1 h can quickly induced *BAG6*, *BAG8*, and *BAG9* and whose transcript levels subsequently reached a maximum after 3 h. Nevertheless, the expression levels of other *BAG*s were not changed or decreased after heat stress (Figure 2). These results suggested that *BAG6*, *BAG8*, and *BAG9* may be important in regulating tomato response to heat stress.

**Figure 3:**
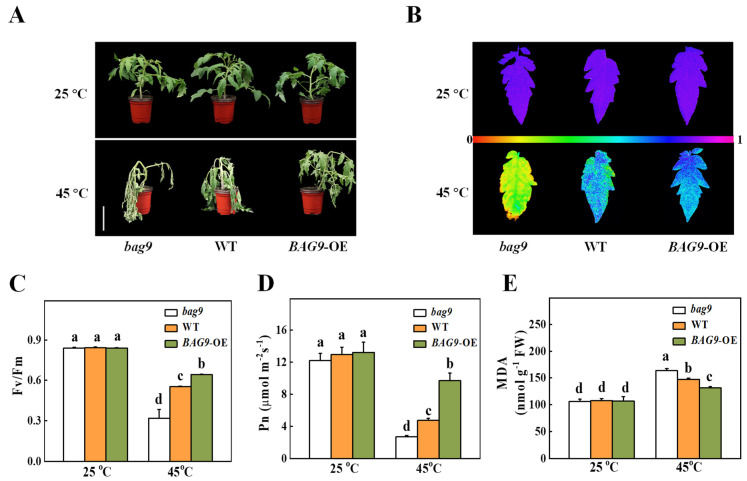
Influence of BAG9 on tomato thermotolerance. (**A**) Representative images of *bag9* mutants, wild type (WT), and *BAG9* overexpressing (*BAG9*-OE) plants without or with heat stress. Bar = 10 cm. The plants were subjected to normal temperature (25 °C) or high temperature (45 °C) treatment for 10 h, photographs of plants were then taken. (**B**,**C**) After undergoing different temperature treatments for 7 h, images of representative leaves showed the maximum photochemical efficiency of photosystem II (*Fv/Fm*). At the bottom, a color gradient showed the strength of the fluorescence signal depicted by each color. (**D**) Net photosynthetic (*P*n) efficiency at 7 h under heat. (**E**) MDA content at 7 h under heat or without heat stress. Data were the means ± SD of three biological replicates. Different letters represented significant differences (*p* < 0.05) according to Tukey’s test.

Then, we analyzed the cis-elements in promoters of *BAG* genes and found that only the *BAG9* promoter contained the heat shock element (HSE), which was transcriptionally regulated by heat shock factors under heat stress (Figure S2). To investigate whether BAG9 was involved in the regulation of plant thermotolerance, we generated the *bag9* mutants and *BAG9* overexpressing plants as described in the “Materials and Methods” section (Figure S1). As shown in Figure 3A, the phenotypes of *bag9* mutants and *BAG9* overexpressing plants were similar to WT plants, when they were grown under normal conditions (Figure 3A). 

To examine how BAG9 functions in tomato under heat, *bag9* mutants, WT plants, and *BAG9* overexpressing plants grown for about 5 weeks were kept in a 45 °C growth chamber for 10 h. The exposure of tomato plants to heat stress resulted in plant withering and decreased *Fv/Fm* value, more significantly in *bag9* mutants compared with WT plants (Figure 3). In contrast, thermotolerance was significantly increased in *BAG9* overexpressing plants with higher *Fv/Fm* value (Figure 3). Moreover, heat stress inhibited photosynthesis in tomato plants. Net photosynthetic rate (*P*n) was decreased by 42.4% in *bag9* mutants but was increased by 100.1% in *BAG9* overexpressing plants compared with WT plants (Figure 3D). Additionally, MDA accumulation was aggravated in *bag9* mutants, while alleviated in *BAG9* overexpressing plants compared with WT plants (Figure 3E). Thus, these results suggested that BAG9 played a positive role in tomato response to heat stress.

### 3.3. BAG9 Alleviates Heat-Stress-Induced ROS Accumulation

**Figure 4:**
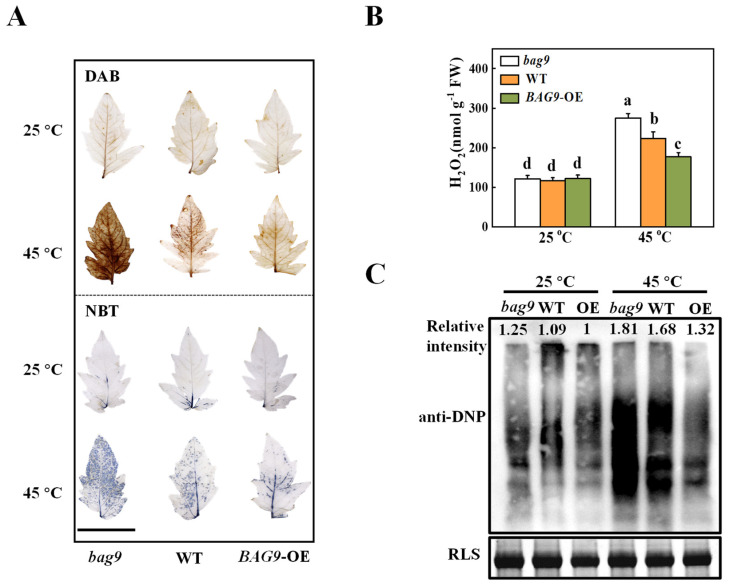
The accumulation of reactive oxygen species (ROS) and oxidative proteins in tomato plants under heat stress. (**A**) Representative images of H_2_O_2_ and O_2_^•−^ accumulation were detected by DAB and NBT staining, respectively. Bar = 5 cm. (**B**) Quantification of H_2_O_2_ at 7 h under heat. (**C**) Oxidative proteins. An anti-DNP antibody was used to detect total proteins on SDS-PAGE. Coomassie Blue staining (CBB) was applied to indicate the protein input, and on the top of the image was the relative intensity of oxidative proteins. Three independent experiments were performed with similar results. Data were the means ± SD of three biological replicates. Different letters represented significant differences (*p* < 0.05) according to Tukey’s test. WT, wild type; *BAG9*-OE, *BAG9* overexpressing plants; RLS, Rubisco large subunit.

ROS production and scavenging keep homeostasis balanced in plants under normal conditions [42]. However, this homeostasis will be disturbed after heat-stress exposure [2]. To verify the effect of BAG9 on heat-induced oxidative stress, we first detected H_2_O_2_ and O_2_^•−^ accumulation. Tomato leaves were stained with DAB dye for histochemical detection of H_2_O_2_ and with NBT dye for O_2_^•−^ detection. As shown in Figure 4, heat stress induced H_2_O_2_ and O_2_^•−^ production in the leaves of WT plants. Interestingly, H_2_O_2_ and O_2_^•−^ production was significantly induced in *bag9* mutants, whereas it was reduced in *BAG9* overexpressing plants (Figure 4A). Similarly, the H_2_O_2_ content was quantitatively analyzed in support of the observation that H_2_O_2_ was more accumulated in *bag9* mutants, but significantly reduced in *BAG9* overexpressing plants compared with WT plants (Figure 4B).

To further investigate whether heat-induced oxidative stress caused the oxidation of functional proteins, SDS-PAGE was used to analyze protein oxidation among proteins isolated from total proteins. Figure 4C illustrated that the accumulation of oxidative proteins was similar in *bag9* mutants, WT, and *BAG9* overexpressing plants under normal conditions. Mutants *bag9* and plants overexpressing *BAG9*, however, had increased and decreased levels of oxidative proteins, respectively, compared to wild-type plants. Thus, these results suggested that BAG9 reduced the accumulation of ROS and the oxidation of protein caused by heat.

### 3.4. BAG9 Enhances Antioxidant Capacity under Heat Stress

**Figure 5:**
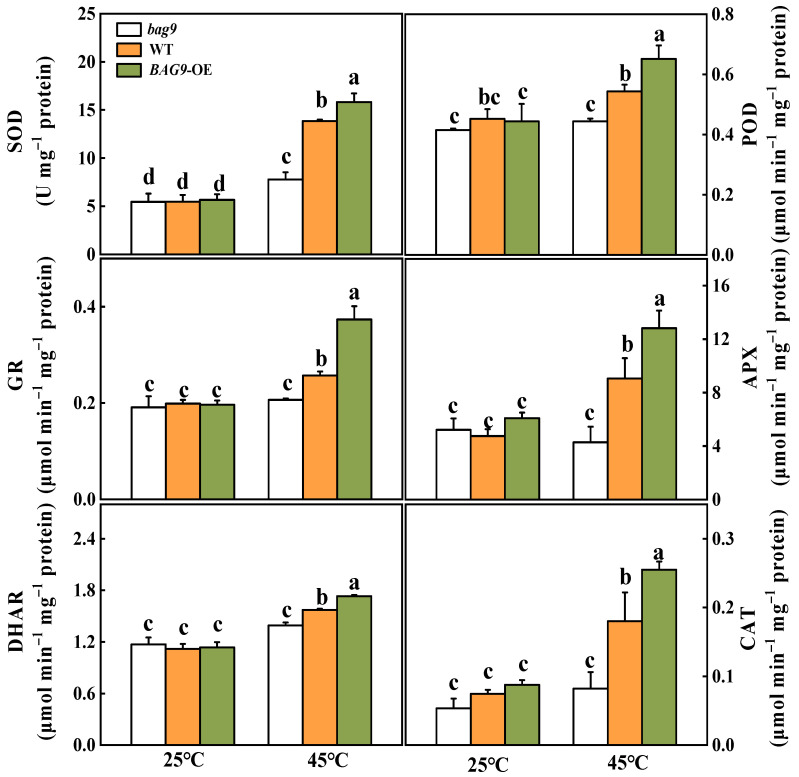
Activities of SOD, POD, APX, GR, CAT, and DHAR with or without heat stress in tomato leaves. Data were the means ± SD of three biological replicates. Different letters represented significant differences (*p* < 0.05) according to Tukey’s test. WT, wild type; *BAG9*-OE, *BAG9* overexpressing plants.

Antioxidant defense mechanisms contain antioxidant enzymes such as SOD, APX, GR, CAT, DHAR, POD, and antioxidants such as ASA and GSH to trap and scavenge free radicals and ROS, thereby protecting plant cells and organelles from destruction and increasing stress resistance [47]. As shown in Figure 5, heat stress increased all six antioxidant enzyme activities in WT and *BAG9* overexpressing plants. However, in *bag9* mutants, POD, APX, GR, DHAR, and CAT activities between control and heat treatment showed no significant difference (Figure 5). The enzyme activities in *BAG9* overexpressing tomato were higher than those in WT. According to these results, BAG9 promoted the activities of antioxidant enzymes under heat stress.

**Figure 6:**
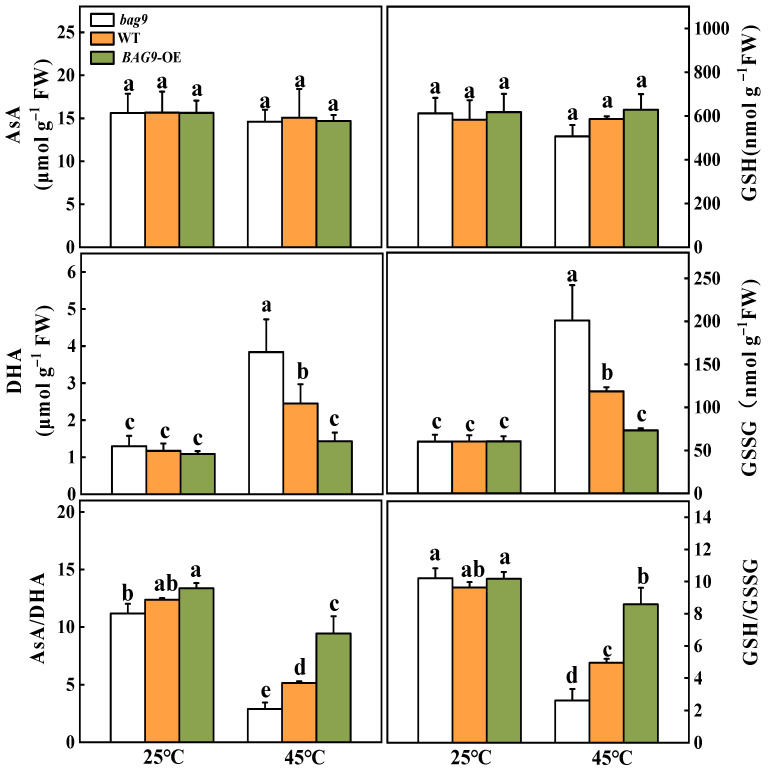
Effects of heat stress on AsA and GSH pools in tomato leaves. Data were the means ± SD of three biological replicates. Different letters represented significant differences (*p* < 0.05) according to Tukey’s test. WT, wild type; *BAG9*-OE, *BAG9* overexpressing plants.

To determine whether BAG9-induced thermotolerance was related to the state of cellular redox, the variation of contents and ratios of AsA/DHA and GSH/GSSG were examined (Figure 6). Heat stress had little effect on the AsA and GSH levels but significantly increased the DHA and GSSG contents, leading to significant declines in the AsA/DHA and GSH/GSSG ratios in all plants compared with control. Under heat stress, the DHA and GSSG contents were considerably increased in *bag9* mutants but reduced in *BAG9* overexpressing plants compared with WT plants. Meanwhile, ratios of AsA/DHA and GSH/GSSG were lower in *bag9* mutants but higher in *BAG9* overexpressing plants compared with WT plants (Figure 6).

### 3.5. BAG9 Interacts with Hsp20s and Maintains Hsps Stability under Heat Stress

**Figure 7:**
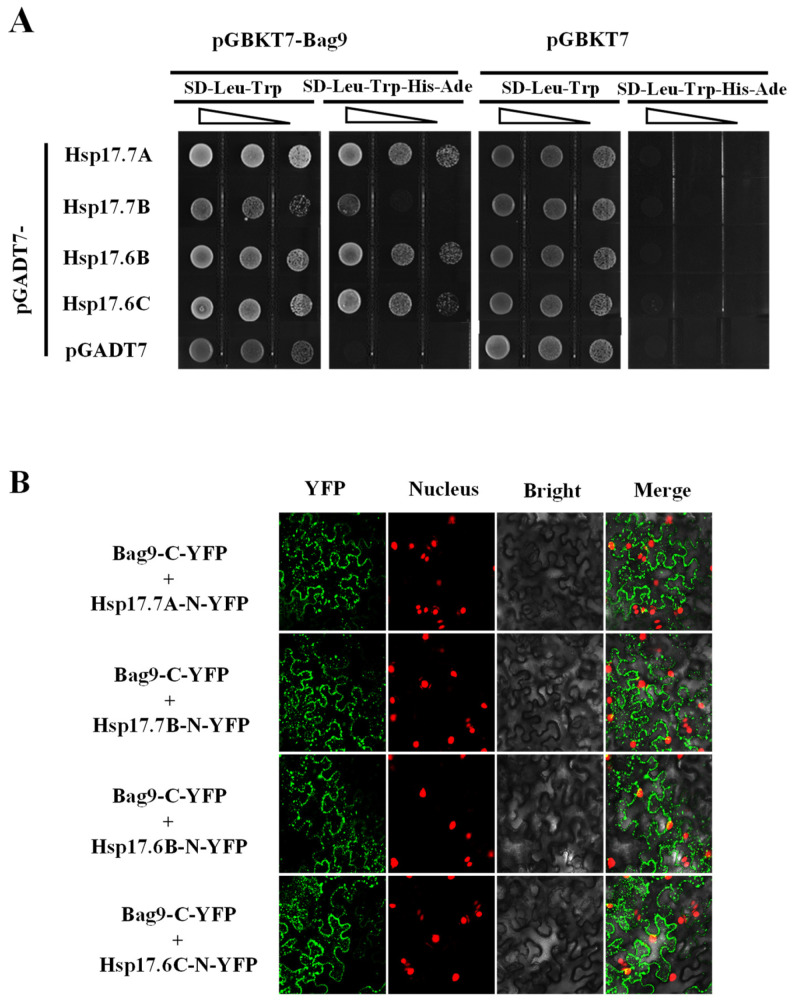
BAG9 interacted with Hsp20s. (**A**) Yeast two-hybrid assay showed interactions between BAG9 and Hsp17.7A, Hsp17.7B, Hsp17.6B, and Hsp17.6C. By growing yeast cells at different concentrations lacking Trp (T), Leu (L), Ade (A), and His (H), the interaction of proteins has been evaluated. (**B**) BiFC analysis showed that the interaction between BAG9 and Hsp20s took place in the cytoplasm. Spliced YFP fusion constructs were transiently coexpressed in *N. benthamiana* leaves for 2 d. The YFP fluorescence signals were obtained by confocal microscopy.

We next identified BAG9-interacting proteins by applying yeast two-hybrid screens. Choosing the fused BAG9 protein as baits, we screened 6 × 10^6^ independent transformants of a tomato cDNA prey library and identified more than twenty clones. The proteins encoded by these positive clones included four Hsp20s (Hsp17.7A, Solyc06g076520; Hsp17.7B, Solyc09g015020; Hsp17.6B, Solyc06g076560; Hsp17.6C, Solyc06g076570). Then, we performed yeast two-hybrid assays to explore whether BAG9 interacted with Hsp20s. By co-transforming the bait and prey vectors, we found that BAG9 interacted with four Hsp20 proteins in yeast (Figure 7A).

To determine whether BAG9 and Hsp20s interact in vivo, we performed a BiFC assay in *A. tumefaciens-infiltrated* tobacco. BAG9 was fused to the C-YFP vector (BAG9-C-YFP) and Hsp20s were fused to the N-YFP vectors (Hsp 17.7A, Solyc06g076520; Hsp17.7B, Solyc09g015020; Hsp17.6B, Solyc06g076560; Hsp17.6C, Solyc06g076570). When the BAG9-C-YFP was co-expressed with four Hsp-N-YFP in tobacco leaves, YFP signals were observed in tobacco cells that had been transformed (Figure 7B). All these experiments revealed that BAG9 interacted with four Hsp proteins.

**Figure 8:**
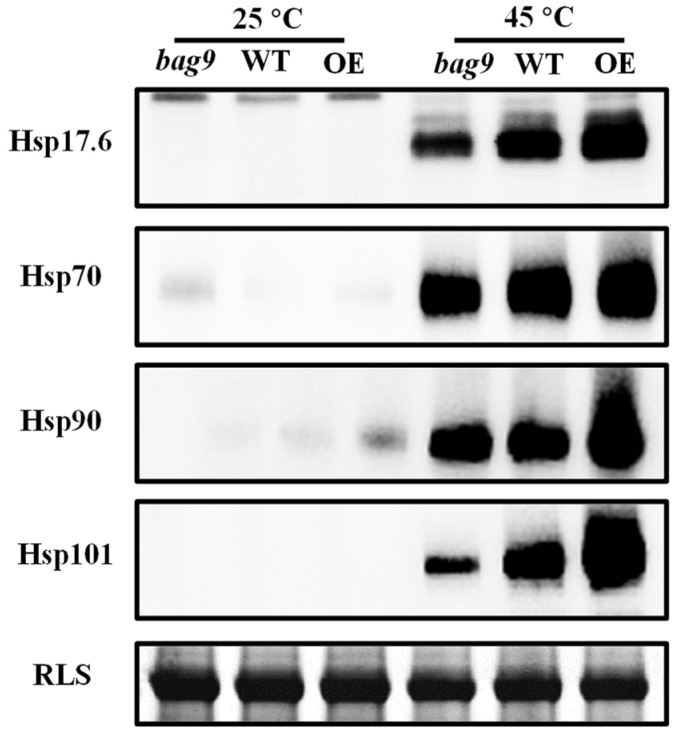
The accumulation of Hsps with or without heat stress in tomato leaves. Hsp17.6, Hsp70, Hsp90, and Hsp101 were detected by immunoblot analysis. After exposing to heat for 7 h, the leaf samples were obtained for experiments. The protein input was indicated by Coomassie Blue staining (CBB). Three independent experiments were performed with similar results. WT, wild type; *BAG9*-OE, *BAG9* overexpressing plants; RLS, Rubisco large subunit.

BAG9 and Hsps are both chaperones. To investigate whether BAG9 affects the stability of Hsps under heat stress, we examined the accumulation of Hsps by Western blotting. As shown in Figure 8, there was almost no difference in the accumulation under normal conditions. While heat stress induced a great accumulation of Hsp20, Hsp70, Hsp90, and Hsp101 in all genotypes. However, compared with WT, the accumulation of these four Hsps was still lower in *bag9* mutants, while higher in *BAG9* overexpressing plants (Figure 8). Thus, BAG9 promoted the stability of Hsps under heat stress.

## 4. Discussion

In this study, we found that the expression of *BAG9* was highly induced under heat stress in tomato. *Bag9* mutants reduced thermotolerance while overexpressing *BAG9* increased thermotolerance as reflected by antioxidant assays. We also found that BAG9 interacted with Hsp20 proteins in vitro and in vivo. Overexpressing *BAG9* enhanced the accumulation of Hsp proteins induced by heat, while the mutants had the opposite tendency. Thus, BAG9 played a crucial role in response to heat stress by regulating cellular redox homeostasis and the stability of heat shock proteins.

Similar to our study, the transcript levels of *OsBAGs* and *BAG* family members in grapes were significantly increased under heat exposure [48,49]. Considering that *BAG9* contained the HSE in the promoter region, it was selected to conduct further research for its potential significance in thermotolerance. BAG9 contained a conserved BAG domain and a CaM binding motif. The BAG domain combined with Hsc70 for decomposing incorrectly folded or translocated chloroplast proteins in *Arabidopsis* [50]. The phylogenetic analysis revealed that BAG9 was most close to OsBAG5, OsBAG6, AtBAG5, and AtBAG6. According to previous research and evolutionary relationships, we speculated that BAG9 may function in temperature protection, especially heat stress by binding with Hsps and maintaining cellular stability or involving in the Ca^2+^ sensing [28,48].

Various kinds of BAG proteins functioning in plant thermotolerance have been identified [28,29]. Heat shock-induced gene 1 (HSG1), a grape Bcl-2-associated athanogene, enhanced heat tolerance and activated *CONSTANS* (CO) expression in transgenic *Arabidopsis* plants [51]. In *Arabidopsis*, heat shock transcription factor (HsfA2) directly bound to HSE motif of *AtBAG6*, which dramatically increased its relative expression under heat stress [52]. AtBAG2 enhanced survival under heat by clearing ROS in plants [28]. AtBAG7 played a key role in mediating the heat-induced UPR pathway [29]. Studies in the BAG family showed that BAG9 stimulated burning symptoms under heat and reduced the thermotolerance of tomato, which did not occur in our experiment [53]. By overexpressing *BAG9* in *Arabidopsis*, the sensitivity to water scarcity, salinity, as well as ABA during the germination of seeds and the growth of seedlings were increased [54]. *BAG5b* (Solyc10g084170, namely *BAG9*) in leaves was activated by various adversity stimuli (extreme temperatures, salinity, and UV light) as well as treatment with phytohormones. Specifically, it improved the resistance to dark-induced leaf senescence by eliminating ROS and downgrading genes associated with leaf senescence [34].

In this study, *P*n, a typical indicator of photosystem I (PSI), was decreased in *bag9* mutants but was highly increased in *BAG9* overexpressing plants compared with WT plants. Similarly, *BAG9* overexpressing plants showed higher *Fv/Fm* values, and the mutants showed compromised *Fv/Fm* values than WT plants. Our results indicated that BAG9 promoted the stability of photosynthesis under heat exposure. Photosynthesis is a thermosensitive physiological process since the photochemical reactions and the carbon metabolism are susceptible to damage under heat exposure [55]. The disruption of the thylakoid membranes inhibits the rate of photosynthesis and PSII activity is also greatly reduced or even stopped under heat stress [56]. Chaperones protect and enhance photosynthesis under stressful environments [56]. The thermal resistance of photosystem II is upregulated by constitutive overexpression of a small Hsp, which suggests that sHsps prevent the damaging of photosynthetic apparatus from high temperature [57]. Hsp90 in the chloroplast was also an irreplaceable chaperone for protein translocating from the membrane into the organelles and served a significant role in heat resistance in photosynthetic organisms [58,59]. Similar to previous research, BAG9 served as a chaperone protein that may protect photosynthesis as shown in this study.

In previous studies, ROS is used as an indicator of plant resistance [60]. Overexpressing *AtBAG4* into the rice and exposing it to osmotic stress revealed that ROS accumulation was significantly reduced in its overexpressing plants [61]. The mutants *Atbag2* and *Atbag6* also showed higher ROS levels and less survival after heat treatment than WT [28]. Similar to the previous study, our results showed that *BAG9* overexpressing plants accumulated less ROS (H_2_O_2_, O^2•−^) and less protein carbonylation (which is a hallmark of protein oxidation), indicating a better resistance to high temperature. MDA is one of the products of ROS-induced membrane damage, whose amount represents the degree of cell membrane lipid peroxidation [60]. The continuous accumulation of MDA is positively correlated to high temperatures [62]. This study discovered that *BAG9* overexpressing plants showed less accumulation of MDA than WT, which indicated that BAG9 may protect biomembrane from being damaged under heat stress.

To mitigate elevated ROS-induced damage, plants have established a well-organized antioxidant-defense mechanism [62]. Antioxidants in plants have been classified into two main types: enzymatic and nonenzymatic antioxidants. The significant antioxidant enzymes in plant cells contain SOD, CAT, POD, and so on [63]. GSH and AsA are vital nonenzymatic antioxidants in plants. Meanwhile, APX, DHAR, and GR serve as significant enzymes in the AsA-GSH cycle [64]. Antioxidants are involved in multiple plant abiotic stresses, including heat stress [63]. Treating seedlings of *Broussonetia papyrifera* at high temperature, the activities of SOD, POD, and CAT were significantly increased [65]. The antioxidant enzyme activities in *Cruciferae* were closely related to high temperature, since its SOD, CAT, and GR activities under high-temperature (32 °C) stress were all higher than those of the control plants (20 °C) [66]. In *Brassica napus*, the developed activities of MDAR, DHAR, and GR under sub-high-temperature treatment (30 °C) elevated the levels of AsA and GSH, resulting in enhanced thermotolerance [67]. Similarly, our results illustrated that *BAG9* overexpressing plants upregulated the activities of antioxidant enzymes (SOD, CAT, POD, APX, DHAR, GR) and ratios of AsA/DHA and GSH/GSSG. All results indicated that the higher thermotolerance in *BAG9* overexpressing plants was probably achieved by enhanced activities of various antioxidants.

Hsps exist widely in plants to prevent stress from inducing damage to cells [68]. Previous studies showed that Hsp70 functioned in a chaperone cycle by Hsp70 chaperone systems [20]. BAG family is a kind of NEF that establishes direct interactions with the ATPase domain of Hsp70 [25]. In tomato, results showed that BAG1 and BAG2 interacted with Hsp70 protein [69]. 

However, there have been no other Hsp–BAG interactions reported. In this study, we discovered that BAG9 interacted with Hsp20s (Hsp17.7A, Hsp17.7B, Hsp17.6B, Hsp17.6C) in the cytoplasm. Hsp20 is the predominant and most abundant class of proteins in many species induced by heat stress [70]. High temperature significantly induced the upregulation of *TaHsp17.4*, *TaHsp17.7A*, *TaHsp19.1*, and *TaHsp23.7* in wheat [71]. *OsHsp20* overexpressing plants had longer root length and higher germination rates than the control under heat and showed better resistance to high temperature [70]. Nonetheless, how BAG9 works under heat stress by interacting with Hsp20s requires further study.

Our results also witnessed the increase in the accumulation of Hsps (Hsp20, Hsp70, Hsp90, Hsp101) in *BAG9* overexpressing plants, indicating that BAG9 stimulated Hsps for enhancing thermotolerance. Hsp90 bound with Hsp70, establishing multiple complexes of chaperones and functioning well in sense signaling [72]. Hsp101, the most functional member in Hsp100, not only increased heat tolerance but also helped in recovery from heat shock [15]. However, the co-operations of BAG9, Hsp90, and Hsp101 need to be studied further.

## 5. Conclusions

In conclusion, we identified that BAG9 was involved in tomato thermotolerance. *BAG9* was highly induced under high temperature. *bag9* mutants were sensitive, while *BAG9* overexpressing plants were resistant under heat stress compared with WT. By analyzing the antioxidant and photosynthetic systems, we found that overexpressing *BAG9* may help in the removal of ROS and protect photosynthesis under heat stress. BAG9 interacted with Hsp20 proteins and protected Hsps accumulation under heat stress. In a word, BAG9 was probably significant for thermotolerance by regulating cellular redox homeostasis and the stability of heat shock proteins. Our findings further illustrated the functions of *BAGs* in adversity modulation, especially temperature stress.

## Data Availability

Data is contained within the article and Supplementary Materials.

## Supplementary Materials

The following supporting information can be downloaded at: https://www.mdpi.com/article/10.3390/antiox11081467/s1, Figure S1: Identification of *BAG9*-OE plants and *bag9* mutants; Figure S2: Analysis of *cis*-acting elements in the promoter region of *BAGs*; Table S1: Gene information of BAG family in tomato; Table S2: Primer sequences designed for RT-qPCR assays; Table S3: Primer sequences designed for pGBKT7 vectors construction; Table S4: Primer sequences designed for pGADT7 vectors construction; Table S5: Primer sequences designed for p2YC and p2YN vectors construction.
