# Supplementary material for: BAG9 Confers Thermotolerance by Regulating Cellular Redox Homeostasis and the Stability of Heat Shock Proteins in Solanum lycopersicum
Source: Antioxidants (Basel). 2022 Jul 27;11(8):1467. doi: 10.3390/antiox11081467 (PMC9404849; doi:10.3390/antiox11081467)
Supplement: Supplementary file 1 [file antioxidants-11-01467-s001.zip › Supplementary Figures.pptx]

## Slide 1
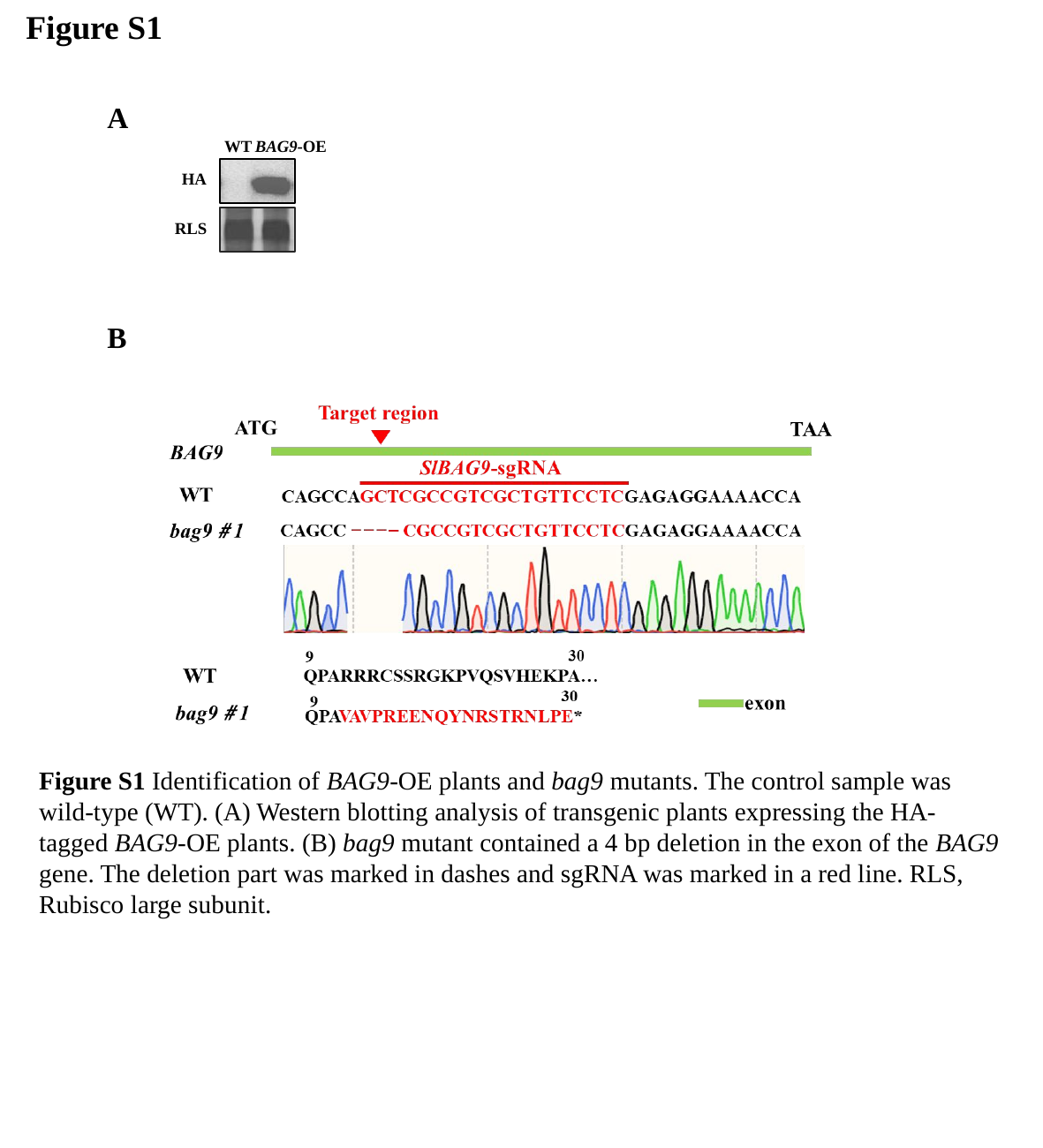

Figure S1
A
BAG9-OE
WT
HA
RLS
B
Figure S1 Identification of BAG9-OE plants and bag9 mutants. The control sample was wild-type (WT). (A) Western blotting analysis of transgenic plants expressing the HA-tagged BAG9-OE plants. (B) bag9 mutant contained a 4 bp deletion in the exon of the BAG9 gene. The deletion part was marked in dashes and sgRNA was marked in a red line. RLS, Rubisco large subunit.

## Slide 2
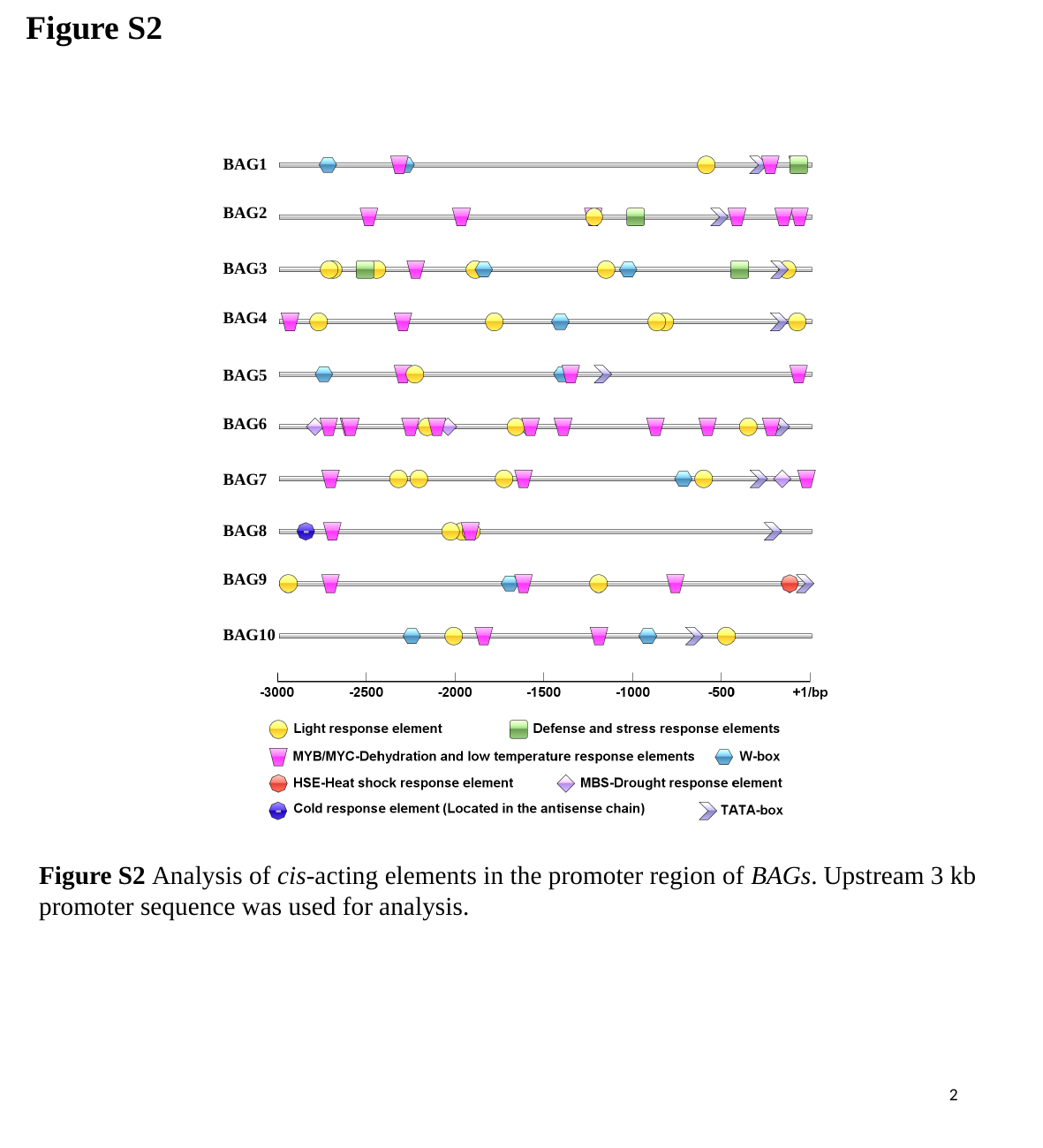

Figure S2
BAG1
BAG2
BAG3
BAG4
BAG5
BAG6
BAG7
BAG8
BAG9
BAG10
Figure S2 Analysis of cis-acting elements in the promoter region of BAGs. Upstream 3 kb promoter sequence was used for analysis.
2
