# Supplementary material for: BAG9 Confers Thermotolerance by Regulating Cellular Redox Homeostasis and the Stability of Heat Shock Proteins in Solanum lycopersicum
Source: Antioxidants (Basel). 2022 Jul 27;11(8):1467. doi: 10.3390/antiox11081467 (PMC9404849; doi:10.3390/antiox11081467)
Supplement: Supplementary file 1 [file antioxidants-11-01467-s001.zip › Supplementary Tables.pdf]

Table S1 Gene information of BAG family in tomato

Gene name	Accession number	CDS	Amino acids	Molecular weight(kDa)	pI
SlBAG1	Solyc03g026220	1026	341	38.253	9.52
SlBAG2	Solyc06g035720	1002	333	37.562	9.45
SlBAG3	Solyc08g080320	855	284	32.331	9.73
SlBAG4	Solyc10g085290	1050	349	37.645	5.03
SlBAG5	Solyc04g014740	300	99	11.512	4.54
SlBAG6	Solyc01g095320	3444	1157	128.578	5.12
SlBAG7	Solyc03g083970	1188	395	45.279	9.43
SlBAG8	Solyc06g072430	1122	373	42.504	5.66
SlBAG9	Solyc10g084170	513	170	19.428	10.26
SlBAG10	Solyc06g007240	867	278	31.528	6.28

Table S2 Primer sequences designed for RT-qPCR assays

Gene name	Accession numbers	Forward primer 5′-3′ sequences	Reverse primer 5′-3′ sequences
SlBAG1	Solyc03g026220	TTCGCCTACACCAGCGAAAC	CCTGGTGCGGGATCAAATGT
SlBAG2	Solyc06g035720	GCAAACAGCAGCACCAATCG	GGCGCTGGAGTGGAATCAAA
SlBAG3	Solyc08g080320	AAGAATGCAGGTGAAAAGGGT	GCTTCATTGACACTTTGGCAT
SlBAG4	Solyc10g085290	GGGTTGGAAGCCTGAATGCC	CAAGCACGGGTCTAGGAGGA
SlBAG5	Solyc04g014740	CAGTCAGGGAGCTGAGACGA	CGCCACCTCTCTCTTTGCAT
SlBAG6	Solyc01g095320	AGGAACCGGTGGATGGAAGT	TGGTCATCTGCTAGACCGAGT
SlBAG7	Solyc03g083970	TGATGGTCCGATCTGCAAAG	GGCACCATCTGGCATATCAA
SlBAG8	Solyc06g072430	CACGATGTAGCACCGCTGAA	TCGAGCATTGGACAGTCCGT
SlBAG9	Solyc10g084170	GGCGGAGTTGATTCGGAGAG	AGCTGCCGGAAACAATGGAG
SlBAG10	Solyc06g007240	GCACAAGGGACAGCTAAAGA	TGGGGATTGATTTGGTGCAT
Actin	Solyc04g011500	GCGAGAAATTGTCAGGGACGT	TGCCCATCTGGGAGCTCAT

The genes are identified from the Sol Genomics Network (http://solgenomics.net/).

Table S3 Primer sequences designed for pGBKT7 vectors construction

Gene name	Accession numbers	Forward primer 5′-3′ sequences	Reverse primer 5′-3′ sequences
SlBAG9	Solyc10g084170	atggccatggaggccgaattcATGGAGAATCTCTTCAATTGGTCC	ccgctgcaggtcgacggatccGCTGCCGGAAACAATGGAG

Table S4 Primer sequences designed for pGADT7 vectors construction

Gene name	Accession numbers	Forward primer 5′-3′ sequences	Reverse primer 5′-3′ sequences
Hsp17.7A	Solyc06g076520	gccatggaggccagtgaattcATGTCTCTGATCCCAAGAATTTTCG	cagctcgagctcgatggatccACCAGAGATCTCAATGGACTTGACC
Hsp17.7B	Solyc09g015020	gccatggaggccagtgaattcATGTCTCTTATTCCAAGCTTCTTTGG	cagctcgagctcgatggatccACCAGAAATGTCAATGGCCTTC
Hsp17.6B	Solyc06g076560	gccatggaggccagtgaattcATGTCTCTGATCCCAAGAATTTTCG	cagctcgagctcgatggatccACCAGAAATCTCAATGGACTTGACC
Hsp17.6C	Solyc06g076570	gccatggaggccagtgaattcATGTCACTGATCCCAAGAATCTTCG	cagctcgagctcgatggatccACCAGAGATCTCAATGGACTTGACA

Table S5 Primer sequences designed for p2YC and p2YN vectors construction.

Gene name	Accession numbers	Forward primer 5′-3′ sequences	Reverse primer5′-3′ sequences
SlBAG9	Solyc10g084170	atttacgaacgatagttaattaacATGGAGAATCTC TTCAATTGGTCC	actgccacctcctccactagtGCTGCCGGAAACAAT GGAG
Hsp17.7A	Solyc06g076520	atttacgaacgatagttaattaacATGTCTCTGATCC CAAGAATTTTCG	actgccacctcctccactagtACCAGAGATCTCAAT GGACTTGACC
Hsp17.7B	Solyc09g015020	atttacgaacgatagttaattaacATGTCTCTTATTC CAAGCTTCTTTGG	actgccacctcctccactagtACCAGAAATGTCAAT GGCCTTC
Hsp17.6B	Solyc06g076560	atttacgaacgatagttaattaacATGTCTCTGATCC CAAGAATTTTCG	actgccacctcctccactagtACCAGAAATCTCAAT GGACTTGACC
Hsp17.6C	Solyc06g076570	atttacgaacgatagttaattaacATGTCACTGATC CCAAGAATCTTCG	actgccacctcctccactagtACCAGAGATCTCAAT GGACTTGACA

The genes are identified from the Sol Genomics Network (http://solgenomics.net/).
